# Complementary Medicine Practice and Use of Dietary Supplements in Pediatric Cancer Patients in Croatia

**DOI:** 10.7759/cureus.30246

**Published:** 2022-10-13

**Authors:** Izabela Kranjcec, Slaven Abdovic, Domagoj Buljan, Nusa Matijasic, Martina Slukan, Jasminka Stepan

**Affiliations:** 1 Oncology and Hematology, Children's Hospital Zagreb, Zagreb, HRV; 2 Pediatrics, Division of Nephrology, Children's Hospital Zagreb, Zagreb, HRV; 3 Oncology and Hematology, Children's Hospital Zagreb, School of Medicine, University of Zagreb, Zagreb, HRV

**Keywords:** herbal medicine, dietary supplements, complementary therapies, children, chemotherapy

## Abstract

Introduction: The use of complementary and alternative medicine (CAM) has become a customary practice among pediatric cancer patients worldwide. The frequency of its use by pediatric cancer patients in Croatia has not been previously determined.

Methodology: In order to establish the frequency and detect factors affecting the use of CAM, a single-center, observational, cross-sectional study was conducted at the Children's Hospital Zagreb during a two-year period. The patients' parents and caregivers were offered an anonymous, multi-item questionnaire that collected socio-economic and clinical data, as well as details on CAM and dietary supplement practice.

Results: Almost half of the participants reported CAM and more than two-thirds reported dietary supplement consumption, predominantly in the intensive phase of the treatment. Factors regarding children or parents had no effect on CAM and supplement utilization. Herbal medicine, vitamins, and minerals were among the most commonly used items. Every 10th child experienced at least one adverse event during CAM and supplement use.

Conclusion: Awareness of the CAM and dietary supplement application in pediatric oncology patients needs to be raised due to the potential interactions with conventional treatment modalities. For this reason, it is extremely important to inform parents and caregivers of pediatric oncology patients about the use of CAM and dietary supplements to predict and mitigate the occurrence and intensity of the side effects. In the majority of our cases, the patient's guardians informed the healthcare professionals about the CAM and supplement implementation in their children, therefore, they were offered additional information about the possible negative impact of CAM and supplement use on standard medical care in a timely manner.

## Introduction

The use of CAM, defined by the National Cancer Institute (NCI) as practices and medical products that are not included in standard medical care, has become a customary practice among pediatric cancer patients worldwide [1]. More than a decade ago almost half of the pediatric cancer patients received CAM, with global prevalence ranging from 20% to 60% [2-3].

The striking differences in CAM use relate predominately to geographic region and country's economic status, as a higher incidence of CAM consumption has been reported in low-middle income countries (LMIC) [4]. However, even among high-income countries (HIC) in Europe slight variability in CAM administration has been noted; its frequency ranges from 35% in German to 42.4% in Dutch or 57% in Irish surveys [5-7]. 

A few potential benefits of CAM have been identified, both physical and psychological. Ameliorating fatigue, balancing sleep patterns, or providing family members with a sense of involvement are some examples. Another advantage has been observed in the setting of end-of-life care, with CAM offering hope where conventional therapy fails to [2, 8].

However, substantially more disadvantages have been well recognized. Potential supplement-conventional drug interactions, treatment-related toxicities, and compliance to standard therapy have raised most concerns [4]. Moreover, with growing data on conventional oncology therapeutic modalities, consulting patients and family members about CAM utilization for most healthcare professionals still presents quite a challenge [8]. 

The incidence of CAM consumption in Croatia varies from 15% in the general population to 54.6% among healthy university students [9-10]. While the prevalence of CAM use among Croatian adult oncology patients was reported as 35%, no studies on pediatric cancer patients in Croatia have been conducted so far [11]. In order to determine the frequency and factors affecting its use, an observational single-center study was designed.

## Materials and methods

An observational, cross-sectional study on the use of dietary supplements and CAM practice among pediatric cancer patients was conducted at the Department of Oncology and Hematology at the Children's Hospital Zagreb. From February 1st 2019 to March 31st 2021, parents and carers of children and adolescents and young adults (AYA) 0- 21 years, diagnosed with a solid tumor or hematologic malignancy, were offered a 29-item anonymous self-administered questionnaire in various stages of treatment (intensive first-line chemotherapy, maintenance, intensive second-line, palliative care) or follow-up (therapy completed). Patients with non-malignant conditions were excluded from the study. 

One hundred and twenty-six subjects were enrolled by signing the informed consent, two participants were excluded due to the irregularly or incompletely filled questionnaires. Non-probability sampling methods were used and participants were selected based on availability and willingness to take part (convenience sampling). Socio-economic and clinical data were collected (patient's age and sex, tumor type, year of diagnosis, treatment phase, parents' age and level of education, religious affiliations, and region of residence). The questions on dietary supplement consumption and alternative therapy use (variety of products, method of procurement and informing, familiarity with positive and negative impacts, cooperation with medical staff -- primary oncologists) were asked. 

Descriptive statistics were used to describe the demographic and disease-related data for study participants. Data distribution has been checked with the Smirnov-Kolmogorov test. Where the assumption was not upheld, nonparametric tests were used. The Mann-Whitney U test was used to compare continuous variables and 𝛘2-test to analyze differences in categorical variables between patient groups. The p-values less than 0.05 were considered statistically significant. Statistical analysis was performed using jamovi (version 1.8.4; the jamovi project, Sydney, Australia).

This study was approved by the Ethics Committee of the Children’s Hospital Zagreb (approval number: 02-23/24-4-1-19) and was conducted according to the Declaration of Helsinki.

## Results

Enrolled participants were treated for malignancy from 2000 to 2021. Table 1 shows a descriptive analysis of patients' demographic and disease-related variables. 

**Table 1:** Descriptive analysis of patients’ demographic and disease-related variables.

	Participants (N=124)
Diagnosis, number (%)	
Leukemia/lymphoma	38 (30.6)
CNS tumor	14 (11.3)
Neuroblastoma/nephroblastoma	21 (16.9)
Osteosarcoma/Ewing’s sarcoma	23 (18.5)
Soft tissue tumor (rabdomiosarcoma)	(6.5)
Other	20 (16.1)
Age categories (years), number (%)	
<1	8 (6.5)
1-4	44 (35.5)
5-9	24 (19.4)
10-14	18 (14.5)
15-21	30 (24.2)
Males, number (%)	69 (56.1)
Year when diagnosed, median (IQR)	2018 (5)
Current treatment phase, number (%)	
Intensive phase	36 (29.3)
Maintenance therapy	20 (16.3)
Relapse	6 (4.9)
Palliative treatment	1 (0.8)
Completion of treatment	60 (48.8)
Age of mother, median (IQR)	35 (8)
Age of father, median (IQR)	38 (10.5)
Maternal education level, number (%)	
Elementary school	8 (6.5)
High school	88 (71.5)
Bachelor’s degree	12 (9.8)
Master’s degree	30 (9.8)
Doctorate	2 (1.6)
Paternal education level, number (%)	
Elementary school	11 (8.9)
High school	88 (71.5)
Bachelor’s degree	12 (9.8)
Master’s degree	12 (9.8)
Doctorate	0
Residence - region, number (%)	
City of Zagreb (Capital of Croatia)	37 (29.6)
Central Croatia	45 (36)
Slavonia, Croatia	13 (10.4)
Dalmatia, Croatia	11 (8.8)
Istria, Croatia	5 (4)
Lika and Gorski Kotar, Croatia	4 (3.2)
Bosnia and Herzegovina	10 (8.0 )
Religion, number (%)	
Catholic	108 (89)
Catholic-Atheist	1 (0.8)
Catholic-Islam	5 (4.1)
Protestant	1 (0.8)
Islam	2 (1.7)
Atheist	4 (3.3)

There were 55 participants (44.4%) who reported implementing CAM (Table 2).

**Table 2:** Statistically significant differences in variables between participants with and without the history of CAM use.

	Using CAM N=55	Not using CAM N=69	p
Diagnosis			0.716
Leukemia/lymphoma	9 (22)	29 (34.9)	
CNS tumor	5 (12.2)	9 (10.8)	
Neuroblastoma/nephroblastoma	8 (19.5)	13 (15.7)	
Osteosarcoma/Ewing’s sarcoma	8 (19.5)	15 (18.1)	
Soft tissue tumor (rabdomiosarcoma)	4 (9.8)	4 (9.8)	
Other	7 (17.1)	13 (15.7)	
Age categories (years), number (%)			0.843
<1	2 (4.9)	6 (7.2)	
1-4	17 (41.5)	27 (32.5)	
5-9	8 (19.5)	16 (19.3)	
10-14	4 (9.8)	14 (16.9)	
15-21	10 (24.4)	20 (24.1)	
Males, N	23	46	1
Year when diagnosed, median	2016	2018	0.03
Current treatment phase, categories			0.614
Intensive phase	11 (27.5)	25 (30.1	
Maintenance therapy	5 (12.5)	15 (18.1)	
Relapse	1 (2.5)	5 (6.0)	
Palliative treatment	0	1 (1.2 %)	
Completion of treatment	23 (57.5)	37 (44.6)	
Age of mother, median	34	36	0.465
Age of father, median	37.0	38.5	0.27
Maternal education level, number %			0.248
Elementary school	1 (2.5)	1 (1.2)	
High school	18 (45)	50 (59.5)	
Bachelor’s degree	7 (17.5)	9 (10.7)	
Master’s degree	13 (32.5)	17 (20.2)	
Doctorate	1 (2.5)	1 (1.2)	
Paternal education level, number (%)			0.719
Elementary school	4 (10)	7 (8.4)	
High school	26 (65)	62 (74.7)	
Bachelor’s degree	5 (12.5)	7 (8.4)	
Master’s degree	5 (12.5)	7 (8.4)	
Doctorate	0	0	
Residence - region, number (%)			0.622
Zagreb	10 (23.8)	27 (32.5)	
Central Croatia	16 (38.1)	9 (34.9)	
Slavonia	4 (9.5)	9 (10.8)	
Dalmatia	6 (14.3)	5 (6)	
Istria	2 (4.8)	3 (3.6)	
Lika and Gorski Kotar	2 (4.8)	2 (4.8)	
Bosnia and Herzegovina	2 (4.8)	8 (9.6)	
Religion, number (%)			0.28
Catholic	36 (87.8)	72 (90)	
Catholic-Atheist	1 (2.4)	0	
Catholic-Islam	3 (7.3)	2 (2.5)	
Protestant	0	1 (1.3)	
Islam	1 (2.4)	1 (1.3)	
Atheist	0	4 (5)	

Patients who were diagnosed with malignancy more recently were significantly in favor of not using CAM (the median year 2018 vs. 2016, p=0.038), even when weighted for cancer site, current treatment phase, the current age of the child and parents, and parental academic level. The most common reported modes of CAM were herbal medicine (36, 65.5%), homeopathy and aromatherapy (12, 21.8%), energy medicine and therapeutic touch (8, 14.5%), yoga and meditation (3, 6.0%), and acupuncture and chiropractic care (2, 3.6%). Eight (14.5%) participants did not report the use of CAM to the medical staff, and five of them reported using energy medicine and therapeutic touch. 

For 50 (90.9%) participants who were using CAM, parents reported they were aware of possible adverse reactions, and seven of them (12.7%) reported having at least one adverse reaction. Eighty-four percent of parents were convinced of the positive effect of CAM on the prescribed treatment course. A total of 109 (87.0%) participants reported the use of food supplements (Table 3). 

**Table 3:** Statistically significant differences in variables between participants with and without the history of using dietary supplements. IQR, inter-quartile range; CNS, central nervous system

	Using supplements N=109	Not using supplements N=15	p
Diagnosis			0.179
Leukemia/lymphoma	31 (28.9)	7 (41.1)	
CNS tumor	16 (14.9)	0	
Neuroblastoma/nephroblastoma	16 (14.9)	5 (29.4)	
Osteosarcoma/Ewing’s sarcoma	19 (17.8)	4 (23.5)	
Soft tissue tumor (rabdomiosarcoma)	8 (7.5)	0	
Other	17 (15.9)	1 (5.9)	
Age categories (years), number (%)			0.013
<1	5 (4.7)	3 (17.6)	
1-4	40 (37.4)	4 (23.5)	
5-9	23 (21.5)	1 (5.9)	
10-14	12 (11.2)	6 (35.2)	
15-21	27 (25.2)	3 (17.6)	
Males, N (%)	57 (52.8)	12 (75)	0.095
Year when diagnosed, median (IQR)	2018 (5)	2018 (2)	0.467
Current treatment phase, categories			0.618
Intensive phase	31 (29.2)	5 (29.4)	
Maintenance therapy	15 (14.1)	5 (29.4)	
Relapse	5 (4.7)	1 (5.9)	
Palliative treatment	1 (0.94)	0	
Completion of treatment	54 (50.9)	6 (35.3)	
Age of mother, median (IQR)	35 (8)	35 (5)	0.499
Age of father, median (IQR)	38 (10.5)	38 (5)	0.726
Maternal education level, number (%)			0.298
Elementary school	7 (6.5)	1 (5.9)	
High school	55 (51.4)	13 (76.5)	
Bachelor’s degree	16 (14.9)	0	
Master’s degree	27 (25.2)	3 (17.6)	
Doctorate	2 (1.9)	0	
Paternal education level, number (%)			0.032
Elementary school	11 (10.4)	0	
High school	68 (64.2)	17 (100)	
Bachelor’s degree	12 (11.3)	0	
Master’s degree	15 (14.2)	0	
Doctorate	0	0	
Residence - region, number (%)			0.005
Zagreb	36 (33.3)	1 (5.9)	
Central Croatia	37 (34.3)	8 (47.1)	
Slavonia	11 (10.2)	2 (11.8)	
Dalmatia	11 (10.2)	1 (5.9)	
Istria	5 (4.6)	0	
Lika and Gorski Kotar	4 (3.7)	0	
Bosnia and Herzegovina	4 (3.7)	5 (34.8)	
Religion, number (%)			0.202
Catholic	95 (91.3)	13 (76.5)	
Catholic-Atheist	1 (0.9)	0	
Catholic-Islam	4 (3.8)	1 (5.8)	
Protestant	1 (0.9)	0	
Islam	1 (0.96)	1 (5.9)	
Atheist	2 (1.9)	2 (11.8)	

**Figure 1:**
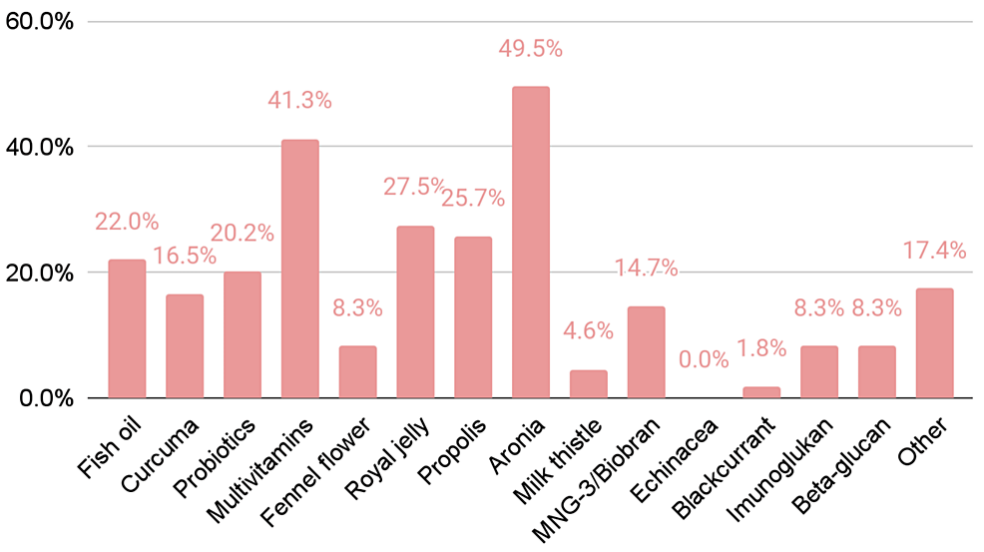
Reported ratio of participants who used a particular dietary supplement (N=109).

Figure 1 shows the reported number of participants who used a particular supplement.

Most commonly, supplements were purchased in pharmacies (80, 78.4%), and rarely via webshops (10, 9.8%). In 98 cases (89.9%), the medical staff was informed about the use of supplements. 

For 78 (71.6%) participants, parents reported they were aware of possible adverse reactions, and 12 of them (11.0%) reported having at least one adverse reaction while using supplements. Only one participant with reported use of supplements had a concomitant adverse reaction while using CAM. Seventy-eight percent of parents were convinced of the positive effect of supplements on the prescribed treatment course. 

**Figure 2:**
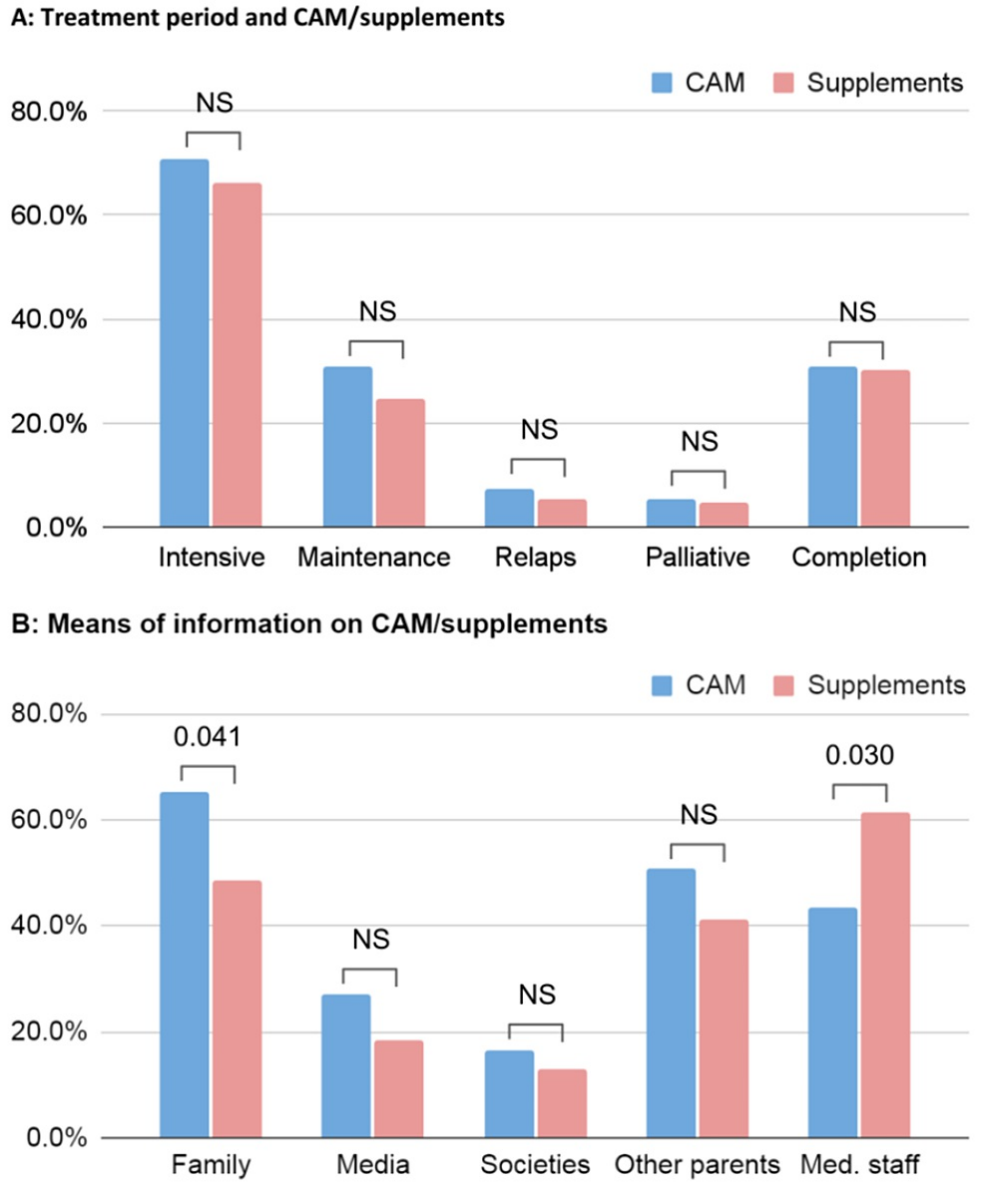
Comparison between the frequency of CAM and supplement usage with regard to the cancer treatment period (A) and sources of information (B). CAM, complementary alternative medicine

No statistical difference was found between the utilization of CAM and supplements during different treatment phases (Figure 2A). Family, medical staff, and parents of other patients were the most common means of information for both CAM and supplements (Figure 2B). In a total of 15 cases, out of 124 (12.1%), neither use CAM nor supplements during their treatment.

## Discussion

This is the first observational study to establish the frequency of the use of CAM and dietary supplements by Croatian pediatric cancer patients. With a frequency of 44.4%, Croatian children with malignancies are slightly less likely to be exposed to CAM than their national adult counterparts (60.3%) [12]. This result is completely comparable to or is slightly below the frequency published in other European and HIC worldwide [5]. In contrast, in regions with reduced financial capabilities or strong cultural predisposition to CAM use reported frequency has been twice as much [13-14]. However, few studies in the last decade are showing a positive association between financial income and CAM use. It is not surprising that wealthier individuals are more inclined to apply alternative treatment modalities as they represent an additional healthcare cost that they cover on their own. 

According to the European literature data, females and parents using CAM themselves were significantly more likely to use CAM in children [6]. In a South American study, mothers' level of education was a significant predictor of CAM use, as was Catholicism affiliation [15]. On the contrary, no disparities in CAM use were observed in our cohort regarding parents' gender, academic level, and religious beliefs. Although hematologic malignancies were the most prevalent cancer type with preschool children and males slightly more represented, no dissimilarities among different tumors, age groups, and gender were detected. 

In the last five years, no change in the prevalence of CAM was observed among French children with cancer as opposed to our results [16]. Namely, CAM usage in our cohort significantly declined over the years. The observed downward trend correlated with the employment of a nutritionist at the department, therefore, it might be a result of the daily expert consultations and directed CAM use. Another possible explanation might be found in the growing trust in Western medicine with rising educational levels nationwide. 

The incidence of CAM use has been reported higher in children and adolescents in whom the frontline conventional therapy has failed, reaching up to 82% [17]. Nevertheless, at the beginning of the last decade, an increase of 11% was documented in CAM use among palliative patients [18]. However, our respondents were most likely to administer CAM to their children during the intensive phase of treatment. Palliative patients were remarkably underrepresented in our cohort, so no conclusion could be drawn on CAM use in the end-of-life context. 

The United State (US) National Center for Complementary and Alternative Medicine (NCCAM) divides CAM therapies into five categories: alternative medical systems, mind-body medicine, biologically based therapies, manipulative and body-based methods, energies (biofield) therapies, and bioelectromagnetic-based therapies [19]. Herbal medicine, homeopathy, and aromatherapy were the three most widely used types of CAM among our participants. This observation can be compared with that of Laengler et al. who noted that homeopathy (therapy with serially diluted remedies) and anthroposophic medicine (therapy with anthroposophic partly highly diluted medicine and non-medical therapies) was applied in 72% of their respondents [5]. Similar results were published by Grootenhuis et al. in the Netherlands, whilst in a small Finnish study, dietary supplements were most commonly used as an adjunct to conventional treatment of pediatric oncology patients [20].

According to NCCAM, CAM also includes biologically based therapies such as herbs and dietary supplements that are commonly used by children with cancer and their families worldwide. These are non-food substances or combination of substances including vitamins, minerals, herbs, plant-based products, and other bioactive compounds taken with the intent of improving health [21]. In our study, the most commonly used dietary supplements were minerals, vitamins and herbal supplements, mostly purchased in pharmacies where parents and caregivers could receive specific information about certain products from pharmacists and other well-trained staff. However, the risk of using dietary supplements lies in their less stringent regulation when compared to other prescribed drugs. In a large number of countries, control of such nutritional supplements by agencies like the European Medical Agency (EMA) or The Agency for Medicinal Products and Medical Devices (HALMED) in Croatia has thus been avoided. This increases the risk of using substances that may, for instance, be contaminated with heavy metals or microorganisms, which increases their toxicity and possible interactions with conventional drugs used in the treatment of pediatric oncology patients [1, 22-23]. 

Even if generally allowed dietary supplements are used, there is always the possibility of adverse events occurring. According to several studies, concomitant use of antioxidants, such as vitamin C or melatonin, and chemotherapeutics, such as cisplatin and doxorubicin, could lead to the neutralization of reactive oxygen species required for the cytotoxic effects of previously mentioned cytostatics [24-26]. As stated in our results, a little more than 10% of our respondents who used dietary supplements or CAM during chemotherapy experienced adverse events, mostly occurring as a mild transient increase in liver transaminases. In previous studies, the side-effect rate was slightly lower, namely, 4% [18]. However, several authors have accentuated the possible severity of the adverse events; e.g. important potential toxicity is the effect of high doses of vitamin E on the coagulation cascade, especially during chemotherapy [27]. 

Therefore, quality communication between parents of a sick child and healthcare professionals is extremely important. According to the study by O'Connor et al., most of their respondents (65%) did not inform their oncologist about the use of CAM [7]. This result is consistent with findings in the general Irish pediatric setting where 54% of participants had not informed their doctor about CAM and dietary supplement use, most likely because they had not been specifically asked about it [28]. With this in mind, our medical team has pointed out to parents and caregivers the importance of reporting the dietary supplements and CAM use, therefore, the rate of treating oncologists being informed about its administration was higher than 80%. Our findings are similar to that of Bold et al. who reported that 71% of the CAM and dietary supplements users in their survey spoke about it with a physician [29].

We are aware of the potential limitations of our survey. Firstly, a single-center design possibly fails to recognize across-country diversities. However, since our Department was declared a Referent center for solid tumors in children and provides medical care for pediatric patients nationwide, our cohort might be considered representative. Secondly, different CAM and supplement definitions and classifications might have been utilized among various surveys, results being, therefore, incomparable. Last but not least, although the majority of parents and carers of pediatric oncology patients confirmed the use of the alternative treatment modalities, honesty in filling out the questionnaire can be considered controversial, as well as whether some other additional alternative preparations were used but not confessed. 

## Conclusions

Pediatric oncology patients are a vulnerable group of individuals in whom the nature of the disease, but also treatment complications instinctively induce the parents to find additional modalities that would cure or alleviate the symptoms of the underlying disease. We are witnessing an increase in the frequency of CAM and dietary supplements use by children with malignancies in the last decade. Therefore, it is the task of amenable healthcare professionals to inform parents and caregivers of pediatric oncology patients about the potential advantages and disadvantages of CAM and dietary supplements and also about possible interactions with conventional cancer treatments. For this reason, it is necessary to raise the awareness of oncologists and other medical staff about the use of CAM and food supplements in order to be able to provide appropriate and adequate information for their patients. Finally, although our research is the first in this region regarding CAM and dietary supplements use by children with malignancies, further prospective research on a wider cohort of children with cancer is certainly needed. What is unexplored today, may prove to be an accepted and effective treatment strategy in the future.
